# Development and Optimization of Edible Antimicrobial Films Based on Dry Heat–Modified Starches from Kazakhstan

**DOI:** 10.3390/foods14112001

**Published:** 2025-06-05

**Authors:** Marat Muratkhan, Kakimova Zhainagul, Kamanova Svetlana, Dana Toimbayeva, Indira Temirova, Sayagul Tazhina, Dina Khamitova, Saduakhasova Saule, Tamara Tultabayeva, Berdibek Bulashev, Gulnazym Ospankulova

**Affiliations:** 1 Department of Food Technology and Processing Products, Technical Faculty, Saken Seifullin Kazakh Agrotechnical Research University, Zhenis Avenue, 62, Astana 010011, Kazakhstan; kamanovasveta@mail.ru (K.S.); bio.dana@mail.ru (D.T.); indira_t85@mail.ru (I.T.); tazhina_saya@mail.ru (S.T.); dina.khamitova@nu.edu.kz (D.K.); saule_aru@list.ru (S.S.); tamara_tch@list.ru (T.T.); berdibek_aruzhan@mail.ru (B.B.); 2 Department of Food Production Technology and Biotechnology, The Engineering–Technological Faculty, Shakarim University, Glinka 20A, Semey 071412, Kazakhstan; zhaynagul.kakimova@mail.ru

**Keywords:** starch-based film, dry heat modification, edible antimicrobial film, biodegradable packaging

## Abstract

This study aimed to design and optimize an edible antimicrobial film incorporating thermally modified starches using a systematic experimental approach. A comprehensive analysis of six starch types—both native and dry heat–modified—was conducted to evaluate their gelatinization clarity, freeze–thaw stability, microstructure (CLSM), and in vitro digestibility. Corn and cassava starches were selected as optimal components based on their physicochemical performance. A series of single-factor experiments and a Box–Behnken design were employed to assess the influence of starch concentration, gelatinization time, glycerol, and chitosan content on film properties including tensile strength, elongation at break, water vapor permeability (WVP), and transparency. The optimized formulation (5.0% starch, 28.2 min gelatinization, 2.6% glycerol, 1.4% chitosan) yielded a transparent (77.64%), mechanically stable (10.92 MPa tensile strength; 50.0% elongation), and moisture-resistant film. Structural and thermal analyses (SEM, AFM, DSC, TGA) confirmed the film’s homogeneity and stability. Furthermore, the film exhibited moderate antioxidant activity and antibacterial efficacy against *Escherichia coli* and *Staphylococcus aureus*. These findings demonstrate the feasibility of using dry heat–modified Kazakhstani starches to develop sustainable antimicrobial packaging materials. However, further studies are needed to explore sensory attributes, long-term storage performance, and compatibility with different food matrices.

## 1. Introduction

In response to increasing global concerns about environmental sustainability and food safety, the development of biodegradable and edible films has gained considerable momentum as a viable alternative to petroleum-based packaging [1,2,3]. Among natural biopolymers, starch stands out due to its abundance, renewability, film-forming capacity, and biocompatibility [4,5,6]. These advantages have led to its widespread use in food, pharmaceutical, and materials industries [7,8].

However, films made from native starches often suffer from critical drawbacks, including poor mechanical strength, high water vapor permeability, and limited functionality, that limit their suitability for active food packaging [9,10,11]. To overcome these limitations, a range of starch modification strategies have been explored, such as chemical derivatization, incorporation of bioactive agents, and physical treatments [12]. Among these, dry heat treatment (DHT) has emerged as a green, food-safe, and cost-effective approach for enhancing starch structure, pasting behavior, and thermal stability, without the use of chemical reagents [13,14,15,16,17].

While extensive work has focused on commercial starches like corn and potato, relatively little attention has been given to starches from underutilized regional crops. Kazakhstan, with its diverse starch-rich crops, including wheat, corn, potato, pea, rice, and cassava, offers a largely untapped resource for biopolymer applications [18,19]. Yet, the high-value utilization of local starches in biodegradable packaging remains poorly investigated, particularly in terms of their physicochemical performance post-modification.

Our previous study systematically evaluated six Kazakhstani starches and demonstrated that dry heat modification significantly improved gelatinization clarity, freeze–thaw stability, and enzymatic resistance, especially for cassava starch, which showed excellent film-forming potential [15]. Despite these promising findings, the synergistic integration of dry heat–modified starches with functional additives to enhance performance and bioactivity in film systems has yet to be explored.

Building on these results, the present study aims to develop a starch-based edible film using dry heat–modified cassava starch, blended with rice starch for improved homogeneity and mechanical strength [20]. To enhance functional properties, glycerol was incorporated as a plasticizer, and chitosan was added for its well-documented antimicrobial and barrier-enhancing effects [21,22,23].

To systematically optimize formulation parameters, a response surface methodology (RSM) based on a Box–Behnken design was employed, evaluating the effects of starch concentration, gelatinization time, glycerol, and chitosan content. The resulting films were comprehensively characterized for their mechanical, thermal, optical, and bioactive properties.

This research addresses a key gap in the development of bioactive edible films using region-specific starch sources and offers a scalable strategy for the sustainable valorization of Kazakhstani crops into high-performance, biodegradable packaging solutions.

## 2. Materials and Methods

### 2.1. Materials

The raw materials were sourced from Kazakhstan, as shown in Table 1. Starch was extracted and cleaned using the water-extraction method described by Liang et al. [24]. The starch modification was based on the method described by Muratkhan et al. [15], with a processing time of 5 h at a temperature of 130 °C. Other lab supplies were purchased from Bio Chem Reagent Co., Ltd., Astana, Kazakhstan. All chemicals and reagents utilized were analytical grades.

### 2.2. Effects of Storage on the Pasting Clarity of Different Starches

The pasting clarity of starches was evaluated according to the method described by Bello-Perez et al. [25] with slight modifications. A 1% (*w*/*v*) starch suspension was prepared by dispersing starch in distilled water under continuous stirring. The suspension was heated in a water bath at 90 °C for 30 min, with stirring every 2 min using a magnetic stirrer to ensure uniform gelatinization. After heating, the samples were cooled to room temperature.

The absorbance of each sample was measured at 650 nm using a UV–Vis spectrophotometer (UV-1800, Shimadzu Corp., Kyoto, Japan) in a 1 cm path-length quartz cuvette. Transparency was expressed as absorbance per centimeter (A/cm), where a lower value indicates higher clarity. The clarity was calculated using the formula:

(1) $$Clarity(\frac{A}{cm})=\frac{A650}{x}$$

where *A*650 is the absorbance at 650 nm and *x* is the path length of the cuvette (1 cm);

To monitor retrogradation effects, the absorbance was further measured after storing the samples at 4 °C for 24, 48, and 72 h.

### 2.3. Determination of Freeze–Thaw Stability of Starch

The freeze–thaw stability of starch was determined according to a standard method. Initially, a 5.0% (*w*/*v*) starch suspension was prepared and heated in a water bath at 95 °C for 30 min with continuous stirring to ensure complete gelatinization.

The resulting starch paste was cooled to room temperature and then frozen at −20 °C for 24 h. Subsequently, the frozen samples were thawed at room temperature for 60 min. After thawing, the samples were centrifuged at 6000 rpm for 20 min, and the weight of the separated supernatant was recorded.

This freeze–thaw cycle was repeated three times, and the degree of phase separation (the amount of water released) was measured after each cycle. The freeze–thaw stability was expressed as the ratio of the weight of the separated supernatant to the initial weight of the starch paste. Each measurement was performed in triplicate, and the mean values were reported [26].

### 2.4. Observation of Starch Granules by Confocal Laser Scanning Microscopy (CLSM)

The morphology of starch granules was observed using a confocal laser scanning microscope (TCS SP8, Leica Microsystems, Wetzlar, Germany) equipped with He/Ne and Ar lasers.

Briefly, 0.5 g of starch sample was accurately weighed and sequentially mixed with 15 μL of 10 mM 8-aminopyrene-1,3,6-trisulfonic acid trisodium salt and 15 μL of 1 M sodium cyanoborohydride solution. The mixture was vortexed and incubated in a water bath at 30 °C for 15 h to allow staining. After incubation, 1.0 mL of deionized water was added, and the sample was centrifuged to remove the excess dye solution.

The stained starch granules were resuspended in a glycerol/water mixture (1:1, *v*/*v*), mounted onto glass slides, and covered with a coverslip. The prepared samples were observed and imaged using the CLSM system [27].

### 2.5. In Vitro Digestibility of Starches

The in vitro digestibility of starches was evaluated based on the method described by Qadir et al. [28] with slight modifications. Briefly, 100 mg of starch sample was accurately weighed into a 50 mL centrifuge tube. Then, 10 mL of 0.5 M sodium acetate–acetic acid buffer (pH 5.2) was added to adjust the sample to an acidic environment.

The mixture was incubated at 37 °C for 10 min. Subsequently, 4 mL of α-amylase solution (3000 U/mL, derived from porcine pancreas) and 1 mL of amyloglucosidase solution (2500 U/mL) were sequentially added.

The reaction system was incubated at 37 °C for 20 min and 120 min, respectively. After each incubation, enzymatic hydrolysis was terminated by boiling the samples in a water bath for 5 min. A 1.0 mL aliquot of the supernatant was diluted 100-fold with deionized water, and the released glucose content was determined using a GOPOD assay kit (Sangon Biotech Co., Ltd., Shanghai, China).

The glucose contents (G20, G120, and F) were expressed in milligrams (mg), and the total starch content (T) was also expressed in milligrams (mg). Based on these values, the contents of rapidly digestible starch (RDS), slowly digestible starch (SDS), and resistant starch (RS) were calculated using the following equations:

(2) $$RDS\left(\%\right)=\frac{\left(G20-F\right)\times 0.9}{T}\times 100,$$

(3) $$SDS\left(\%\right)=\frac{\left(G120-G20\right)\times 0.9}{T}\times 100$$

(4) RS(%)=100−RDS(%)−SDS(%)

where G120 and G20 are the amounts of glucose released after 120 min and 20 min of hydrolysis, respectively;

F is the amount of free glucose initially present;

T is the total starch content.

### 2.6. Preparation of Film-Forming Solutions

Edible films were prepared based on the method of Zheng et al. [29] with slight modifications. Chitosan was dissolved in deionized water to obtain a 0.5% (*w*/*v*) solution. Native starch was then added at a concentration of 1.5% (dry basis) and the mixture was heated in a water bath at 90 °C for 15 min with continuous stirring. Glycerol was subsequently added at a concentration of 1% (*w*/*v*) as a plasticizer. The resulting solution (10 mL per sample) was poured into 7 cm diameter Petri dishes and dried at 30 °C for 12 h. The dried films were then conditioned at 25 °C and 52% relative humidity for 48 h before testing.

### 2.7. Single-Factor Experiments

To evaluate the effects of individual formulation and processing parameters on the properties of the edible starch-based films, a series of single-factor experiments were conducted. In the starch concentration experiment, starch content was varied at 1%, 2%, 3%, 4%, and 5% (*w*/*v*), while glycerol concentration (5%, *w*/*v*) and gelatinization time (30 min) were held constant. To investigate the influence of starch type and ratio, binary mixtures of corn and cassava starch were prepared with a fixed total starch content of 3% (*w*/*v*). The tested corn-to-cassava ratios included 1:0, 1:1, 0:1, 2:1, and 1:2. The effect of gelatinization time was assessed by varying the heating duration at 20, 25, 30, 35, and 40 min, with starch and glycerol concentrations fixed at 3% and 5%, respectively.

The influence of glycerol concentration was examined by adjusting its content to 1%, 2%, 3%, 4%, and 5% (*w*/*v*), while maintaining starch concentration at 3% and gelatinization time at 30 min. Lastly, to optimize chitosan content, concentrations of 0.5%, 1.0%, 1.5%, 2.0%, and 2.5% (*w*/*v*) were tested, while starch (3%), glycerol (5%), and gelatinization time (30 min) were kept constant. All film-forming solutions were prepared under consistent heating conditions at 90 °C for 15 min and poured into 7 cm diameter Petri dishes for drying as described in Section 2.5 [30].

Starch concentration (1–5%): Preliminary trials showed that concentrations below 1% resulted in poor film integrity, while concentrations above 5% led to excessive viscosity, non-uniform casting, and brittle films upon drying. Similar concentration ranges were also reported to be effective for film formation in previous studies [31].

Glycerol content (1–5%): This range was chosen based on its common use as a plasticizer in starch-based films. Low glycerol levels (<1%) resulted in stiff films with reduced flexibility, while high levels (>5%) caused tackiness and reduced mechanical strength [32].

Gelatinization time (20–40 min): Gelatinization time significantly affects starch solubilization and the homogeneity of the film-forming solution. The selected range ensures complete gelatinization without inducing excessive hydrolysis or degradation [33].

Chitosan content (0.5–2.5%): This range was informed by earlier findings indicating that low concentrations may be insufficient for antimicrobial efficacy, while high concentrations affect transparency and homogeneity of the film [34].

### 2.8. Response Surface Methodology (RSM)

Based on the single-factor results, a Box–Behnken design (BBD) was employed to optimize the film formulation. Four independent variables were selected: starch concentration (1–5%), gelatinization time (20–40 min), glycerol concentration (1–5%), and chitosan concentration (0.5–2.5%), each evaluated at three levels. A total of 27 experimental runs were generated to explore linear, interaction, and quadratic effects of these variables on film characteristics [35].

### 2.9. Determination of Film Thickness, Transparency, Water Vapor Permeability (WVP), and Water Contact Angle (WCA)

The thickness of the films was measured at five random locations using a micrometer (Mitutoyo Corporation, Kawasaki, Japan), and the mean value was calculated. Transparency was determined by measuring the absorbance at 600 nm using a UV-Vis spectrophotometer (UV-2600, Shimadzu Corporation, Kyoto, Japan). Samples were cut into 2 × 4 cm strips for analysis.

Water vapor permeability (WVP) was assessed based on a modified method described by Rodríguez et al. [32], under conditions of 25 °C and 52% relative humidity. The water contact angle (WCA) was measured using a sessile drop method with 5 μL distilled water droplets, recorded with an optical contact angle measurement system (OCA 15EC, DataPhysics Instruments GmbH, Filderstadt, Germany). Measurements were performed at five points per film [36].

### 2.10. Measurement of Mechanical Properties of Films

Tensile strength and elongation at break were determined using a universal testing machine (Model 1036PC, KeXin Industrial Instrument Co., Ltd., Shijiazhuang, China) following the ASTM D882 standard [37], with a 50 N load cell, an initial grip separation speed of 20 mm/min, and a stretching speed of 3 mm/min. Five replicates were measured for each sample [38].

### 2.11. Thermal Characterization of Films by Differential Scanning Calorimetry (DSC)

Thermal properties were analyzed using a differential scanning calorimeter (DSC-Q2000, TA Instruments, New Castle, DE, USA). Approximately 3–4 mg of each sample was sealed in aluminum pans and heated from 10 °C to 200 °C at a rate of 10 °C/min under a nitrogen atmosphere (flow rate: 50 mL/min) [39].

### 2.12. Thermal Stability Analysis of Films by Thermogravimetric Analysis (TGA)

Thermal stability was assessed using a thermogravimetric analyzer (TGA, Mettler-Toledo, Greifensee, Switzerland). Samples (~3–4 mg) were heated from 25 °C to 800 °C at a rate of 10 °C/min under nitrogen flow. Weight loss profiles were recorded to evaluate decomposition temperatures [40].

### 2.13. Scanning Electron Microscopy (SEM) Analysis

The samples were prepared according to the method described by Vasques et al. [41]. The morphological structure of the plastic (3 mm × 3 mm) was observed using a scanning electron microscope (SEM) (JCM-7000, JEOL Co., Ltd., Tokyo, Japan). Briefly, the sample was attached to conductive adhesive and coated with gold. For cross-sectional observation, the sample was frozen and fractured, then photographed at a magnification of 3000× and 800×.

### 2.14. Surface Morphology Observation of Films by Atomic Force Microscopy (AFM)

Surface morphology was characterized using an atomic force microscope (AFM, Veeco Instruments Inc., Plainview, NY, USA) operated in tapping mode. Scans were performed at 20 ± 0.5 °C and 49.5 ± 2% relative humidity to analyze surface topography [42].

### 2.15. Evaluation of Film Color Properties by Colorimetry

The color properties of the films were measured using a colorimeter (Konica Minolta CM-700d, Tokyo, Japan) in SCI mode under D65 illumination conditions, with a measurement geometry of C/2°. Five random points were measured per sample, and mean values were reported.

### 2.16. Assessment of Antioxidant Activity of Films Using DPPH and ABTS Radical Scavenging Assays

The antioxidant activity was evaluated using DPPH and ABTS radical scavenging assays. For the DPPH assay, 1 mL of film extract was mixed with 4 mL of 5 × 10^−3^ M DPPH methanol solution, incubated in the dark for 30 min, and absorbance was measured at 517 nm. For the ABTS assay, 1 mL of ABTS solution (adjusted to an absorbance of 0.750 ± 0.025 at 734 nm) was mixed with 100 μL of film extract, and absorbance was measured after 1 min at 734 nm. The radical scavenging activity (%) was calculated for both assays [43,44].

### 2.17. Evaluation of Antibacterial Activity of Films Against Escherichia coli and Staphylococcus aureus

The antibacterial activity was determined against *Escherichia coli* and *Staphylococcus aureus* as per the method described by Goy et al. [45]. The bacterial strains were selected as representatives of Gram-negative and Gram-positive pathogens, respectively. Film extracts (10%, *v*/*v*) were added to LB or NB broth containing bacterial suspensions (10^6^ CFU/mL) and incubated at 37 °C. The growth inhibition was assessed by measuring optical density at 600 nm (OD600), as detailed in previous studies.

### 2.18. Statistical Analysis

All experiments were conducted in triplicate. Data analysis using SPSS Statistics 19.0 (SPSS Inc., Chicago, IL, USA). Results were expressed as mean ± standard error and further analyzed using Duncan’s multiple range tests with a significance level of *p* < 0.05.

## 3. Results

### 3.1. Gelatinization Transparency as an Indicator of Starch Structural Stability

In this study, the gelatinization transparency of various starch types was measured at a wavelength of 650 nm. According to Table 1, starch transparency changed over the storage period, indicating its significance in assessing structural stability and retrogradation behavior.

As reported in the literature, cassava starch is known for producing highly transparent pastes due to its low lipid and phosphorus content, as well as its relatively small and uniform granule structure, which reduce light scattering during gelatinization. Consistent with these findings, cassava starch in our study exhibited the highest transparency after 24 h of storage (0.97 ± 0.08), even though its initial transparency appeared low (0.24 ± 0.01). This initial value may reflect the rapid retrogradation or gel network restructuring that occurred during the cooling process before measurement. In comparison, corn starch (0.76 ± 0.03) and potato starch (0.44 ± 0.05) showed moderate initial transparency, while rice and pea starches exhibited lower values.

Following 24 h of storage, cassava starch exhibited a significant increase in transparency (0.97 ± 0.08), indicating its susceptibility to structural changes during storage and suggesting the occurrence of molecular rearrangements within the gel network. In contrast, potato starch showed a marked decrease in transparency (0.15 ± 0.06), implying retrogradation and reassociation of starch polymers during storage.

At 48 h, a recovery in transparency was observed for potato starch (0.85 ± 0.04), indicating reorganization and alterations in solubility properties following the initial decline. A decrease in transparency for wheat starch (0.29 ± 0.02) and corn starch (0.26 ± 0.07) further confirmed retrogradation phenomena during storage.

After 72 h, an increase in transparency was noted for corn starch (0.65 ± 0.03), reflecting its structural stability. Transparency levels of potato starch (0.49 ± 0.01) and wheat starch (0.36 ± 0.09) remained relatively stable, while the decrease in pea starch transparency (0.23 ± 0.07) suggested the potential for gel network restructuring.

Overall, although wheat starch exhibited the highest initial transparency, its transparency decreased over time due to retrogradation. Cassava starch demonstrated improved transparency during storage, indicating greater stability. Although potato starch initially showed low transparency, it experienced a notable increase after 48 h. As illustrated in Table 2, these findings highlight that the molecular structure, amylose-to-amylopectin ratio, and storage time are key factors influencing the gelatinization transparency of starch.

Studies have shown that starch transparency is primarily influenced by the amylose-to-amylopectin ratio, granule size distribution, and the starch’s capacity for molecular rearrangement following gelatinization [46,47]. For example, starches with small granule sizes—such as tapioca and pea starch—tend to form uniform gel networks after gelatinization, resulting in reduced light scattering and thus an increasing trend in transparency during storage [48]. In contrast, wheat starch, with its relatively high amylose content, accelerates the retrogradation process, leading to a rapid decline in transparency and indicating poorer structural stability [49]. Additionally, Donmez et al. [50] pointed out that hydration and the formation of intermolecular hydrogen bonds during starch gelatinization also contribute to variations in optical behavior. Therefore, the transparency changes observed in this study not only reflect storage stability but also suggest potential differences in the performance of different starches when applied in film formulations.

### 3.2. Comparative Analysis of Freeze–Thaw Stability in Different Starches

In this study, the freeze–thaw stability of different starch types was investigated. According to Table 3, the level of supernatant separation (an indicator of paste breakdown) varied with each cycle, reflecting the starch’s ability to undergo structural reorganization and the degree of retrogradation.

In the first cycle, wheat starch exhibited the highest separation rate (67.04 ± 2.16%), indicating its structural instability during freezing. Corn starch (53.92 ± 3.45%) and pea starch (45.92 ± 1.56%) showed moderate levels of separation. The lowest supernatant levels were observed in rice starch (19.36 ± 2.17%) and potato starch (32.47 ± 2.73%), possibly due to their higher water-binding capacity.

In the second cycle, wheat starch (61.97 ± 4.14%) and pea starch (52.48 ± 3.06%) continued to show signs of instability, indicating structural weaknesses following gelatinization. Meanwhile, potato starch (13.49 ± 2.82%) and rice starch (11.24 ± 3.37%) maintained the lowest levels of supernatant separation, demonstrating superior structural stability.

By the third cycle, a significant increase in supernatant separation was observed for potato starch (59.95 ± 3.43%) and rice starch (28.25 ± 4.86%), suggesting a decline in their structural stability during prolonged freeze–thaw processes. In contrast, wheat starch (22.74 ± 1.68%) and corn starch (20.91 ± 1.26%) exhibited reduced separation levels, which may indicate stabilization of their retrogradation processes.

The phase separation behavior of different starches during freeze–thaw cycles is mainly related to their internal structure and water-binding capacity. A high amylose content typically contributes to the formation of a dense gel network, which helps retain moisture and reduces water release over multiple freeze–thaw cycles [51]. For instance, rice and potato starches exhibit lower syneresis rates, likely due to their good hydration properties and internal hydrophilic structures [52]. In contrast, wheat and pea starches release more water during repeated cycles, suggesting that their gel networks are looser and less capable of maintaining structural stability. Similar phenomena were observed in the study by Vamadevan et al. [53], where starches with higher amylopectin content were more prone to retrogradation and phase separation. Therefore, the results of freeze–thaw stability tests not only reflect the adaptability of starches during storage and processing but also help predict their film performance under fluctuating humidity conditions.

### 3.3. Confocal Laser Scanning Microscopy (CLSM) Observation of Starch Granules

**Figure 1:**
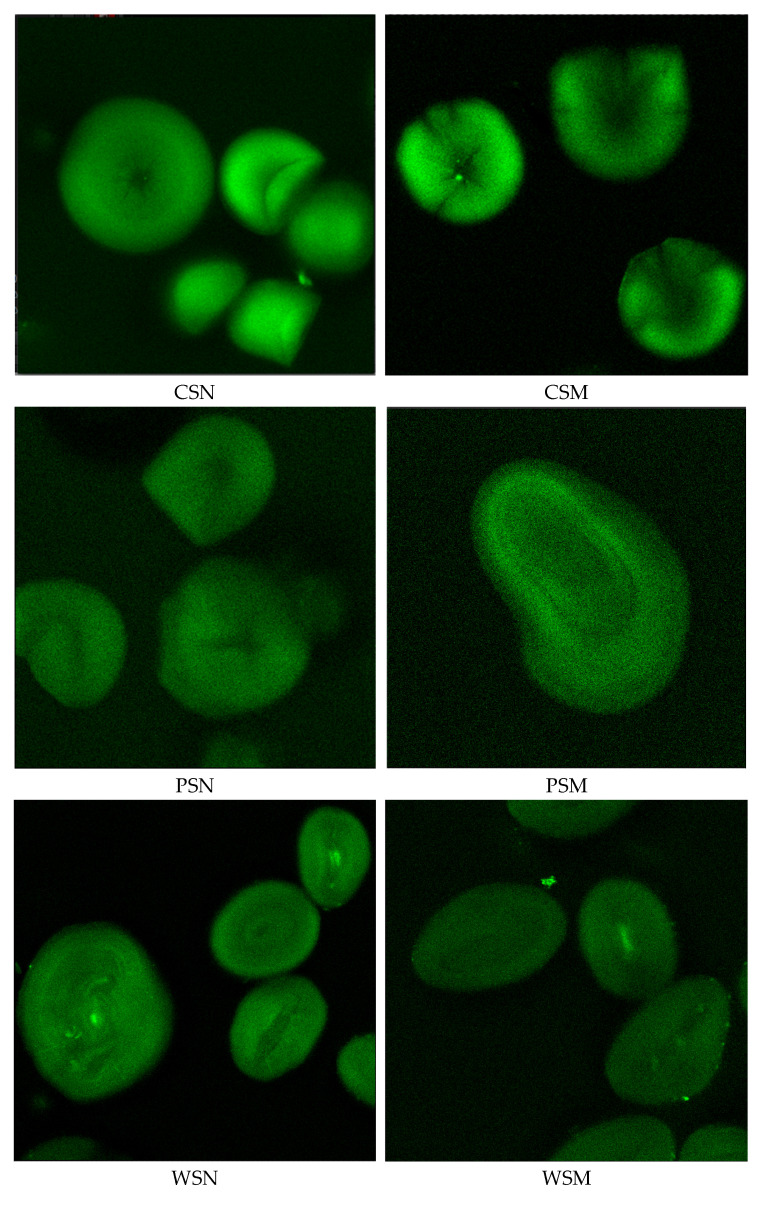

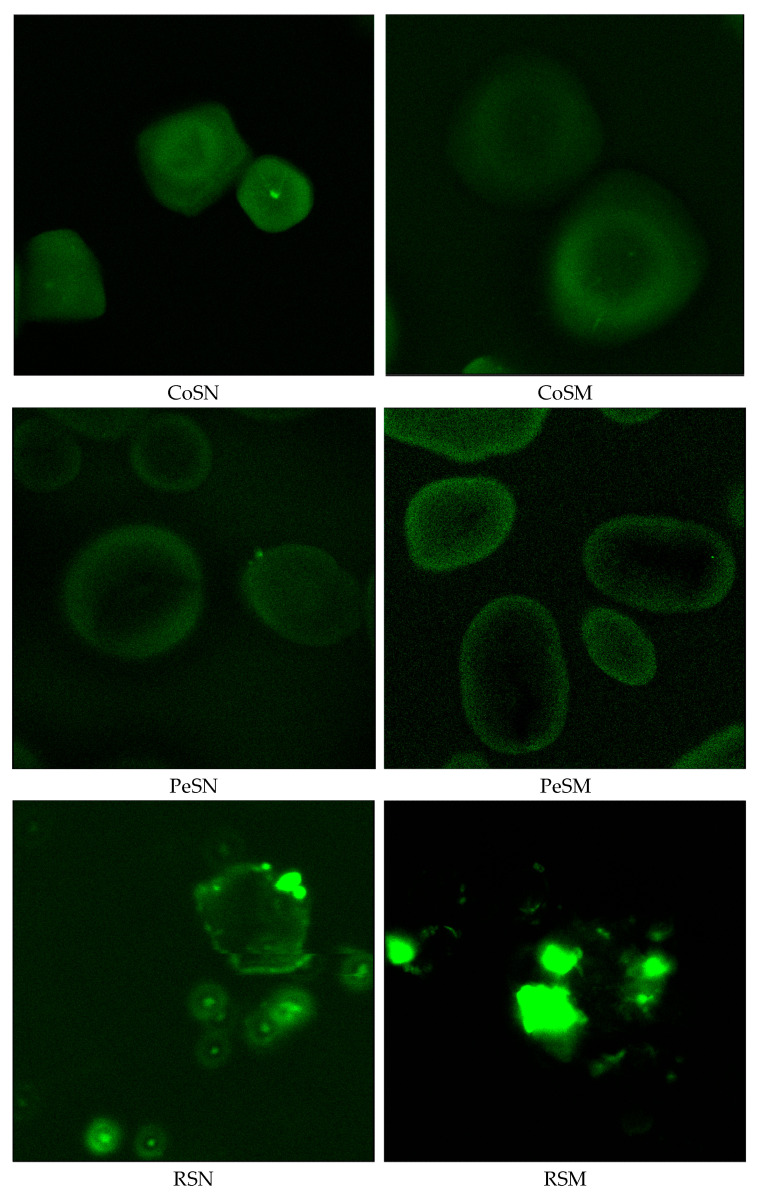
CLSM Images of Various Starches: CSN—cassava native starch samples, CSM—cassava modified starch samples, PSN—potato native starch samples, PSM—potato modified starch samples, WSN—wheat native starch samples, WSM—wheat modified starch samples, CoSN—corn native starch samples, CoSM—corn modified starch samples, PeSN—pea native starch samples, PeSM—pea modified starch samples, RSN—rice native starch samples, RSM—rice modified starch samples.

In this study, confocal laser scanning microscopy (CLSM) was employed to investigate the internal structure and molecular organization of native and thermally modified starches. As shown in Figure 1, thermal treatment had a significant impact on the morphology of starch granules, their light transmission properties, and structural arrangement.

CLSM images of the native starches revealed well-defined granules with uniform light reflectance and high crystallinity. Potato, wheat, and corn starches exhibited strong fluorescence signals, confirming the preservation of their crystalline regions. In contrast, rice and cassava starches displayed weaker fluorescence, indicative of a higher proportion of amorphous structures.

Microscopic images of thermally modified starches showed noticeable structural changes within the granules. Potato starch exhibited reduced light transmittance and structural heterogeneity, possibly reflecting the redistribution of amylose and amylopectin and alterations in the internal architecture. Wheat starch granules showed decreased light refraction and morphological changes, suggesting a reorganization of their molecular arrangement. Corn starch showed disrupted uniformity in light transmission under CLSM, indicating granule restructuring that may enhance molecular interactions during film formation.

For pea and rice starches, CLSM revealed a decline in light refraction and changes in internal granule structure, suggesting higher sensitivity to thermal treatment and potential for structural reorganization during film processing. Cassava starch exhibited high reflectivity, partial granule disintegration, and increased amorphous character, likely associated with reduced thermal stability.

Overall, CLSM results confirmed significant alterations in the structural organization of thermally modified starches. The reduced light transmission observed in potato, corn, and wheat starches may contribute to improved film-forming properties, while the structural reorganization in pea and rice starches could enhance molecular interactions during film development. In contrast, the pronounced disintegration of cassava starch indicates reduced structural stability and limited film-forming capability. These findings verify the morphological transformations of thermally modified starches and highlight their potential applicability as materials for antimicrobial and biodegradable film production.

CLSM images reveal a clear structure–property relationship between starch granule morphology and film performance. Thermal treatment partially disrupts starch granules, promoting molecular rearrangement during gelatinization and resulting in denser, more uniform films [53]. For instance, treated corn and wheat starches show reduced light scattering and blurred granule boundaries, indicating crystalline disruption that supports stable film formation. In contrast, strong reflections and high amorphous content in tapioca starch suggest lower thermal stability and weaker mechanical properties. These findings align with the study by Imran et al. [54], emphasizing that microstructural changes directly affect film transparency, strength, and barrier properties. Thus, CLSM confirms the effects of dry heat treatment and aids in selecting appropriate starch sources for film applications.

### 3.4. In Vitro Digestibility of Native and Thermally Modified Starches

In this study, the in vitro digestibility properties of native and thermally modified starches were assessed by quantifying the levels of rapidly digestible starch (RDS), slowly digestible starch (SDS), and resistant starch (RS). According to Table 3, significant changes in digestibility parameters were observed depending on the starch type and dry heat treatment.

Among the native starches, cassava (52.0 ± 1.5%) and corn (48.0 ± 1.4%) exhibited the highest rapidly digestible starch (RDS) levels, likely due to their high amylopectin content and loosely packed granular structure [25,28]. Potato (45.0 ± 1.6%) and pea (42.0 ± 1.3%) starches showed moderate RDS values, while rice starch had the lowest RDS content (35.0 ± 1.5%), which may be attributed to its dense granule morphology and higher resistance to enzymatic hydrolysis.

Following dry heat modification, the RDS content decreased consistently across all starch types. For instance, modified cassava starch decreased from 52.0% to 40.0 ± 1.3%, and corn starch from 48.0% to 39.0 ± 1.2%, indicating enhanced structural resistance to enzymatic breakdown—possibly due to reorganization of the starch matrix and partial retrogradation during thermal treatment [14,16].

The slowly digestible starch (SDS) content in native starches ranged from 24.0 ± 1.2% to 32.0 ± 1.2%. Potato (32.0 ± 1.2%) and rice (31.0 ± 1.1%) starches demonstrated the highest SDS values, suggesting slower hydrolysis rates by α-amylase. Upon dry heat treatment, SDS levels increased in pea (from 27.0 ± 1.0% to 33.0 ± 1.0%) and wheat (from 28.0 ± 1.0% to 30.0 ± 1.0%), reflecting improved granule stabilization and reduced susceptibility to enzymatic attack [48].

A notable increase in resistant starch (RS) content was observed after dry heat treatment. Native starches showed RS values between 23.0 ± 1.5% and 34.0 ± 1.3%, while their modified counterparts ranged from 31.0 ± 1.3% to 42.0 ± 1.2%. In particular, rice starch increased from 34.0% to 42.0 ± 1.2%, indicating substantial resistance to α-amylase, potentially due to increased crystallinity and the formation of retrograded amylose complexes [46,52].

Overall, these findings confirm that dry heat treatment significantly alters the digestibility profile of starches by reducing the RDS fraction and enhancing RS content. This shift toward slower digestion reflects improved molecular order and enzyme resistance, which are favorable traits for edible packaging applications that require slow-release or structurally stable biopolymer matrices [14,16,48].

### 3.5. Single-Factor Optimization of Edible Antibacterial Film Formulation

#### 3.5.1. Effect of Corn Starch Concentration on Mechanical Properties of the Films

Corn starch was selected as the primary film-forming material due to its favorable balance of initial transparency, freeze–thaw stability, and gel-forming capacity compared to other starches evaluated in this study. As presented in Table 2 and Table 3, corn starch exhibited relatively high initial transparency (0.76 ± 0.03) and moderate freeze–thaw stability—attributes that contribute to the formation of clear, flexible, and mechanically stable films. Additionally, corn starch is widely available, cost-effective, and has a well-established capacity for forming cohesive film networks, making it an appropriate candidate for further development.

**Figure 2:**
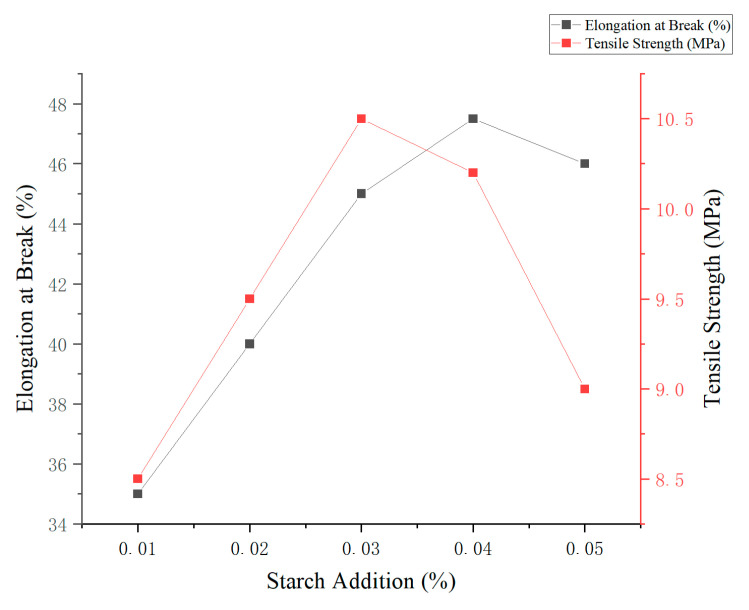
Effect of corn starch concentration on the mechanical properties of edible films.

As shown in Figure 2, both elongation at break and tensile strength increased significantly (*p* < 0.05) with increasing corn starch concentration. The maximum values were observed at 4% starch content, with an elongation at break of 54.47% and a tensile strength of 13.88 MPa. However, further increases in starch concentration beyond 4% led to a significant decline in both mechanical parameters (*p* < 0.05), likely due to excessive solid content interfering with polymer network formation. Based on the comprehensive evaluation of its effects on tensile strength and elongation, a corn starch concentration of 3% was selected for further optimization in subsequent experiments [15].

#### 3.5.2. Effect of Corn-to-Cassava Starch Ratio on Film Performance

**Figure 3:**
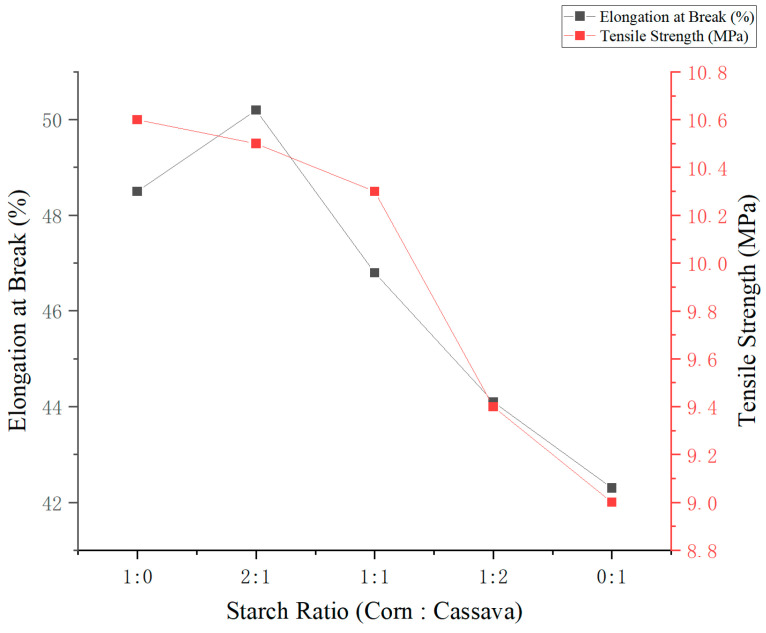
Effect of corn-to-cassava starch ratio on the mechanical properties of edible films.

As shown in Figure 3, varying the ratio of corn starch to cassava starch (at a total concentration of 3%, *w*/*v*) had a significant effect on the mechanical properties of the edible films. With increasing proportions of corn starch, both elongation at break and tensile strength initially increased, reaching a maximum before declining at higher ratios. The film prepared with a 2:1 corn-to-cassava starch ratio exhibited the highest mechanical performance, with an elongation at break of 50.2% and a tensile strength of 10.5 MPa. This suggests that the synergistic interaction between the two starches can significantly enhance both flexibility and strength. In contrast, films composed entirely of cassava starch (0:1 ratio) displayed inferior mechanical properties, confirming the critical role of corn starch in improving film integrity. Therefore, a 2:1 corn-to-cassava ratio is recommended as the optimal formulation for developing biodegradable packaging films with balanced mechanical performance.

#### 3.5.3. Influence of Glycerol Concentration on Flexibility and Strength of the Films

**Figure 4:**
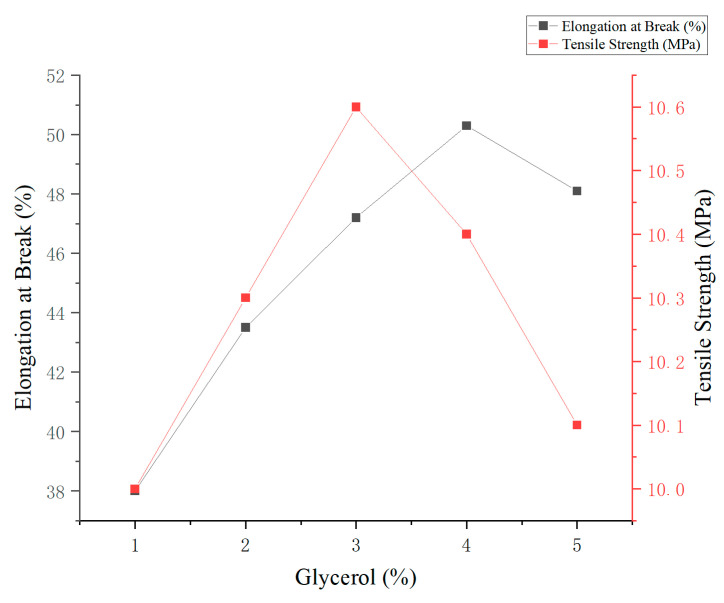
Effect of glycerol concentration on the mechanical properties of starch-based edible films.

This study evaluated the effect of glycerol concentration, used as a plasticizer, on the mechanical properties of edible antimicrobial films prepared from corn and cassava starch. The total starch concentration was fixed at 3% (*w*/*v*), and the gelatinization time was set at 30 min. Glycerol content was varied from 1% to 5% (*w*/*v*), and the resulting changes in elongation at break and tensile strength are presented in Figure 4.

The results showed that increasing glycerol concentration significantly improved the film’s flexibility. The maximum elongation at break (50.3%) was observed at 4% glycerol. Conversely, the highest tensile strength (10.6 MPa) was recorded at 3% glycerol, indicating enhanced structural stability of the film matrix at this concentration. Further increases in glycerol content beyond 4% led to a slight decline in both mechanical parameters, likely due to excess plasticizer disrupting intermolecular interactions within the polymer network.

Overall, glycerol concentrations in the range of 3% to 4% provided an optimal balance between film strength and flexibility. The 3% level was identified as the most suitable concentration for potential application in edible and antimicrobial food packaging materials.

#### 3.5.4. Effect of Gelatinization Time on the Mechanical Properties of Edible Antimicrobial Films

The results indicated that gelatinization time had a significant impact on the mechanical properties of edible antimicrobial films prepared from corn and cassava starch. Under fixed conditions of starch content (3%, *w*/*v*) and glycerol concentration (5%, *w*/*v*), the highest values for elongation at break (50.1%) and tensile strength (10.5 MPa) were obtained at a gelatinization time of 30 min. This duration likely represents the optimal point at which starch undergoes complete gelatinization, resulting in a well-formed polymer network.

**Figure 5:**
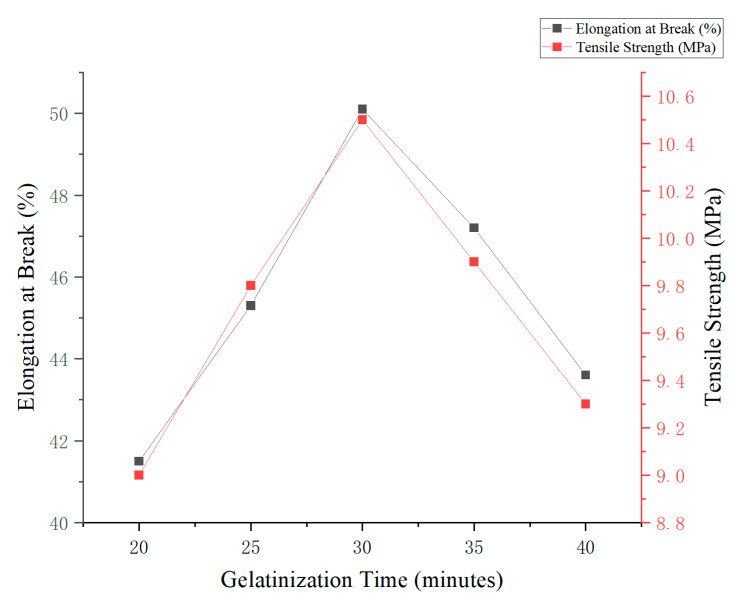
Effect of gelatinization time on the mechanical properties of starch-based edible films.

As shown in Figure 5, extending the gelatinization time beyond 30 min (e.g., to 35 and 40 min) led to a noticeable decline in both elongation and tensile strength. This reduction can be attributed to the degradation of starch chains and the weakening of the polymer matrix due to prolonged thermal exposure.

Therefore, a gelatinization time of 30 min is considered optimal for producing edible antimicrobial films, as it ensures a favorable balance between flexibility, mechanical strength, and structural integrity.

#### 3.5.5. Effect of Chitosan Concentration on Mechanical, Barrier, and Optical Properties of Edible Antimicrobial Films

The effect of varying chitosan concentrations (0.5–2.5%, *w*/*v*) on the physical properties of edible antimicrobial films based on corn and cassava starch was evaluated. The investigated properties included elongation at break, tensile strength, water vapor permeability (WVP), and transparency. All other conditions were kept constant (starch concentration: 3%, glycerol: 5%, gelatinization time: 30 min).

**Figure 6:**
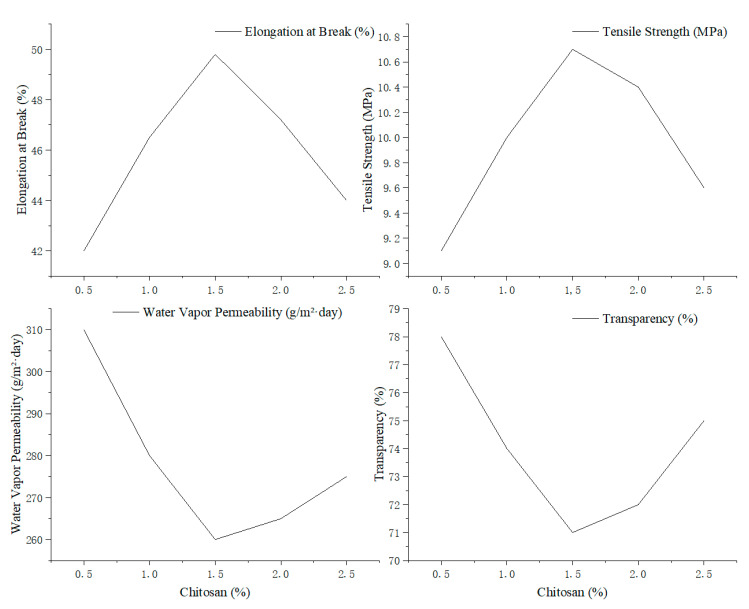
Effect of chitosan concentration on the physical properties of edible starch-based films.

As shown in Figure 6, both elongation at break and tensile strength increased with rising chitosan concentration, peaking at 1.5% with values of 49.8% and 10.7 MPa, respectively. Beyond this point, both properties declined, suggesting that moderate chitosan levels reinforce the film matrix, while excessive amounts may disrupt polymer network uniformity.

Similarly, WVP reached its lowest value (260 g/m^2^/day) at 1.5% chitosan, indicating improved moisture barrier properties. However, transparency gradually decreased with increasing chitosan content, likely due to enhanced light scattering caused by dispersed chitosan polymers in the film matrix.

At last, a chitosan concentration of 1.5% was identified as optimal for achieving a balanced enhancement of mechanical strength, moisture resistance, and acceptable transparency in edible antimicrobial films.

The observed decline in mechanical properties and increased water vapor permeability at chitosan concentrations above 1.5% can be attributed to the disruption of polymer network uniformity. Excessive chitosan introduces an imbalance in the film matrix due to over-saturation of polymer chains, leading to phase separation, poor hydrogen bonding efficiency, and heterogeneous domain formation. This structural irregularity weakens the cohesive forces within the matrix and impairs its ability to effectively resist mechanical stress or block moisture transmission.

Furthermore, chitosan’s cationic nature may cause excessive electrostatic repulsion or aggregation when added beyond the optimal level, preventing proper molecular entanglement with starch and glycerol [35].

### 3.6. Experimental Design Based on Response Surface Methodology (RSM)

To optimize the formulation process for edible antimicrobial films, a response surface methodology (RSM) approach was adopted based on the results of preliminary single-factor experiments. A Box–Behnken design (BBD) was constructed using Minitab Statistical Software (version 18, Minitab LLC, State College, PA, USA) to evaluate the effects of four independent variables: starch concentration (A, %), gelatinization time (B, min), glycerol concentration (C, %), and chitosan concentration (D, %). The coded levels for each factor are shown in Table 4.

The complete Box–Behnken experimental design and measured response values, including tensile strength, elongation at break, water vapor permeability, and transparency, are presented in Table 5.

To optimize the transparency (%) of the edible antimicrobial film, a second-order regression model was developed based on the Box–Behnken design, incorporating four independent variables: starch concentration (A), gelatinization time (B), glycerol concentration (C), and chitosan concentration (D). The full quadratic model included linear, interaction, and squared terms. Despite the lack of statistical significance for most linear terms (*p* > 0.05), the quadratic term A^2^ (*p* = 0.100) had a notable effect, indicating that excessive or insufficient starch levels may reduce film transparency (The detailed ANOVA and related tables are provided in the attachment.).

The regression equation for predicted transparency was as follows:

(5) Ytransparency=73.97+0.350A−0.342B−0.242C+0.117D−1.779AA−0.467BB−0.867CC           −0.429DD−0.85AB+0.52AC−0.88AD+0.60BV+0.02BD−1.05CD

Using grid search optimization across 194,481 simulated conditions, the optimal transparency (77.5%) was achieved under the following coded conditions: A = +1.0 (≈5.0% starch), B = −0.1 (≈28.2 min), C = −0.2 (≈2.8% glycerol), D = −0.1 (≈1.4% chitosan).

**Figure 7:**
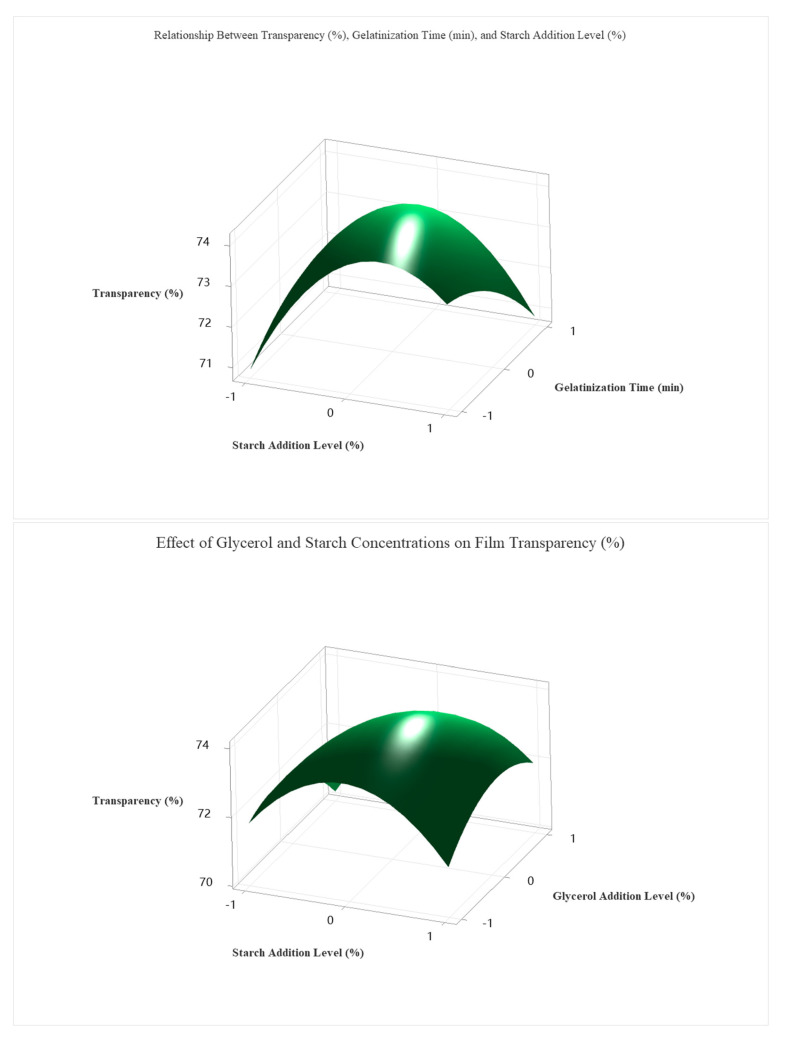

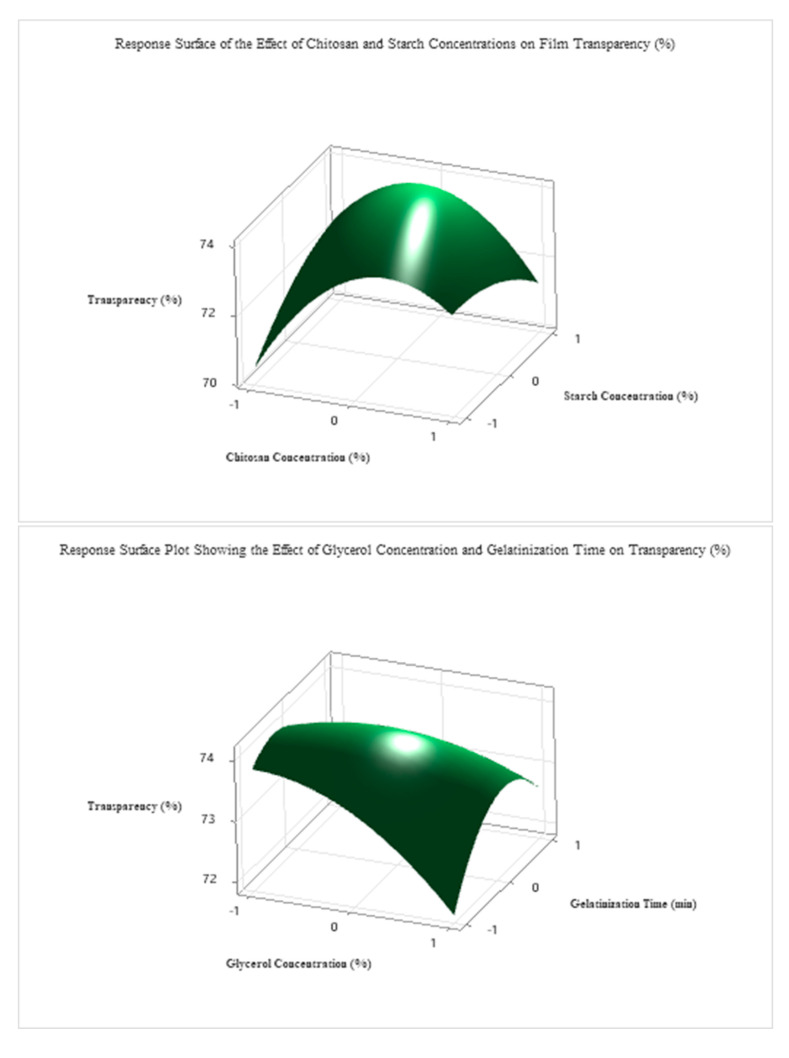

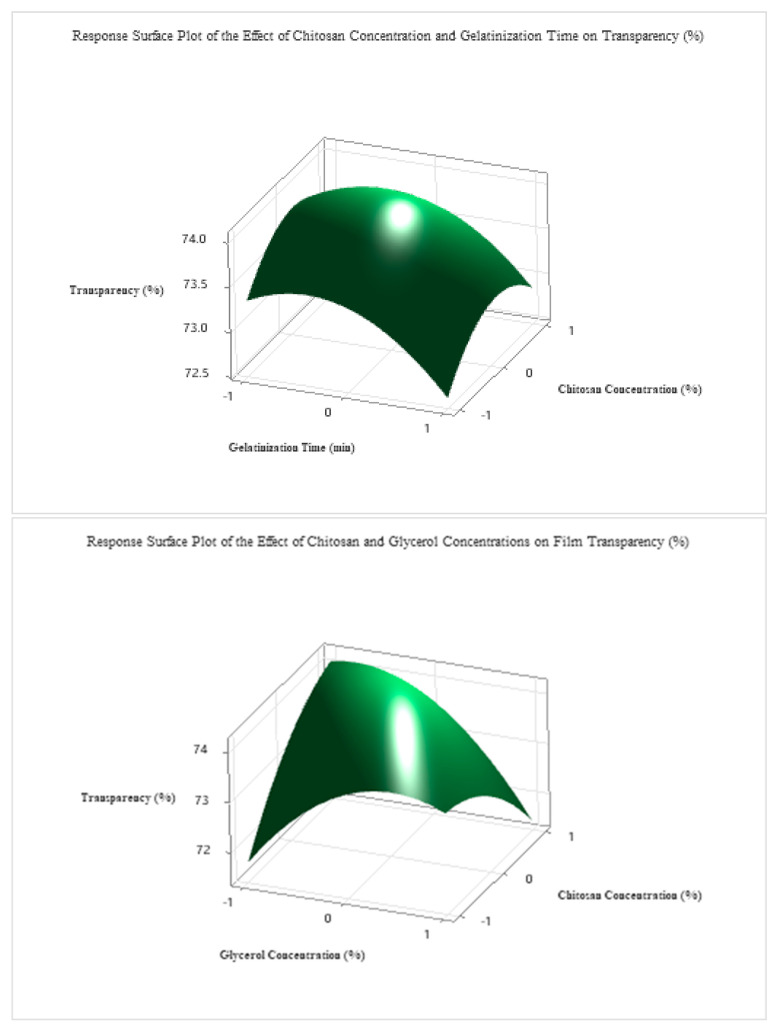
Response surface plots showing the effects of process variables on the transparency of edible antibacterial films.

As shown in Figure 7, these optimal parameters were validated experimentally and resulted in a highly transparent and mechanically stable film. Among the four investigated factors, starch concentration exerted the most significant influence on transparency, followed by glycerol content, gelatinization time, and chitosan concentration.

The coded optimal levels were translated into actual processing parameters, as shown in Table 6. The optimal formulation consisted of 5.0% starch, a gelatinization time of 28.2 min, 2.6% glycerol, and 1.4% chitosan. This composition yielded the highest predicted transparency value (77.5%) and was experimentally confirmed, demonstrating the reliability and predictive accuracy of the developed regression model.

### 3.7. Validation of the Optimized Formulation

To verify the accuracy of the optimized conditions obtained from the response surface methodology, validation experiments were conducted. While keeping all other processing parameters constant, trials were performed in triplicate under the predicted optimal conditions: starch content of 5.0%, gelatinization time of 28.2 min, glycerol content of 2.6%, and chitosan content of 1.4%.

The average transparency (%) achieved under these conditions was 77.64%, which is very close to the model-predicted value of 77.5% and higher than the maximum value observed in all initial experimental groups. This strong agreement confirms the reliability of the regression model in predicting transparency and validates its accuracy. Furthermore, the results indicate that the optimized formulation is feasible for potential application in industrial settings.

### 3.8. Physicomechanical Properties of the Optimized Edible Antimicrobial Film

According to the results presented in Table 7, the edible antimicrobial film formulated based on the RSM-optimized composition (5.0% starch, 28.2 min gelatinization time, 2.8% glycerol, and 1.4% chitosan) exhibited several desirable physico-mechanical and functional properties.

Specifically, the film had a thickness of 0.0492 mm, indicating uniform structure and suitable thinness. Its transparency, represented by the absorbance at 600 nm (A_600_), was measured at 1.205, confirming good visible light transmittance. The calculated water vapor permeability (WVP) was 6.652 × 10^−10^ g/m·s·Pa, demonstrating effective moisture barrier capacity.

Furthermore, the water contact angle (WCA) was 84.6°, indicating moderate surface hydrophobicity. The film showed excellent mechanical performance, with a tensile strength of 10.92 MPa and an elongation at break of 50.0%, reflecting both flexibility and robustness.

### 3.9. Structural and Thermal Properties of the Optimized Edible Antimicrobial Film

**Figure 8:**
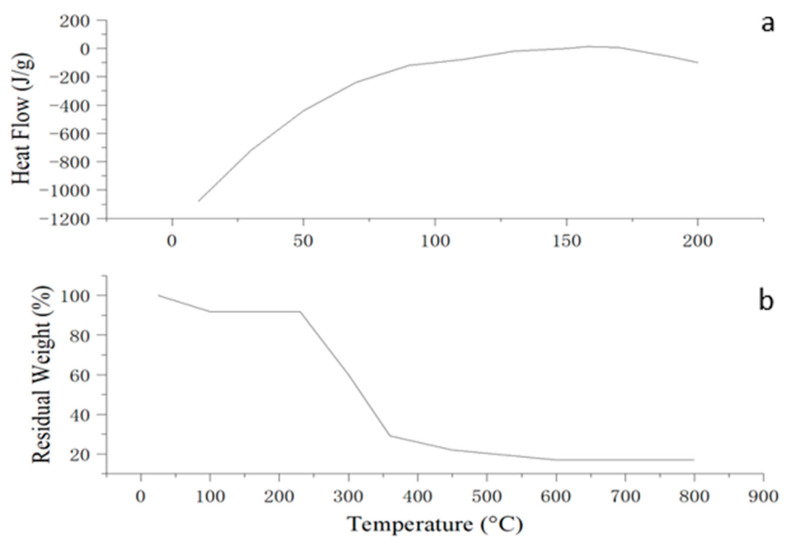
Thermal analysis of the optimized edible antimicrobial film: (**a**) DSC thermogram showing thermal transitions; (**b**) TGA curve indicating thermal stability and degradation profile.

The thermal analyses confirmed that the optimized edible antimicrobial film exhibits favorable thermal behavior for food packaging applications. The DSC thermogram (Figure 8a) displayed a broad endothermic transition centered around 158 °C, which may be attributed to the melting of semicrystalline starch regions or relaxation of amorphous domains within the polymer matrix. Additionally, a glass transition temperature (Tg) of approximately 74 °C was observed, indicating that the film retains structural integrity under typical storage and handling conditions. These thermal transitions suggest acceptable molecular compatibility among the film components and the presence of both amorphous and semicrystalline structures in the matrix.

The TGA thermogram revealed a multi-step degradation profile. The initial weight loss below 120 °C was attributed to moisture evaporation. The main thermal decomposition occurred between 230–360 °C, corresponding to the breakdown of the starch–chitosan matrix and plasticizer components. A residual mass of approximately 17% remained at 800 °C, indicating the presence of thermally stable residues. These results demonstrate that the film is thermally stable up to around 230 °C, supporting its potential for use in food packaging applications that do not involve high-temperature exposure.

### 3.10. Microscopic Structural Analysis of the Optimized Film (SEM)

**Figure 9:**
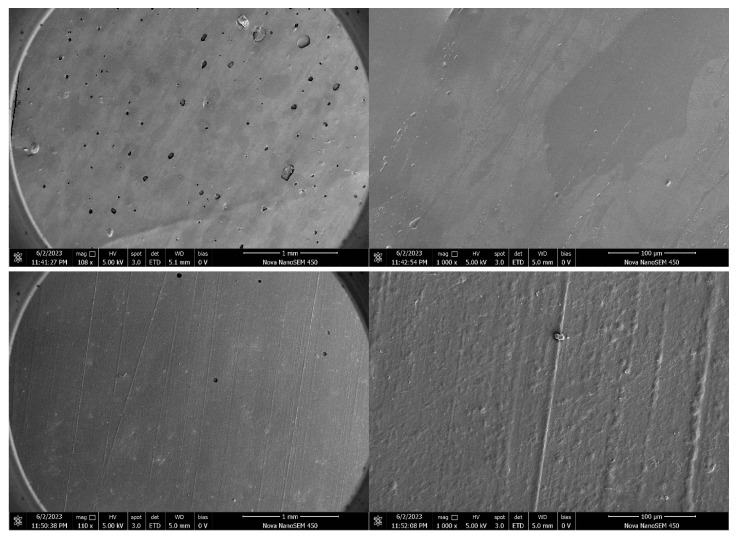

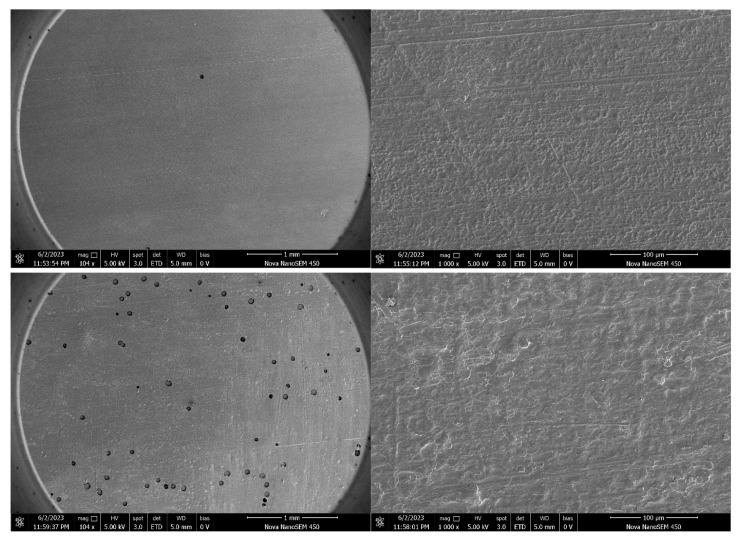
SEM micrographs of the optimized edible antimicrobial film, showing uniform, dense, and continuous surface morphology with micron-level homogeneity.

Microscopic examination of the optimized edible antimicrobial film revealed a uniform, dense, and continuous surface morphology (Figure 9). Scanning electron microscopy (SEM) images showed a smooth film surface with homogeneously distributed particles at the micron scale. The films prepared under optimized conditions displayed a clearly defined amorphous or semi-crystalline microstructure, which supports their consistent mechanical and barrier properties.

These structural characteristics are indicative of a well-formed polymer network that contributes to the film’s performance under practical conditions. The presence of semi-crystalline domains may enhance strength and flexibility, while the amorphous regions contribute to film transparency and homogeneity.

At last, the SEM analysis confirmed that the optimized film exhibits a high-quality microstructure, thermal stability, and functional properties aligned with application requirements. These findings support its potential as an eco-friendly material for use in food packaging.

SEM observations of continuous and compact structures are closely linked to improved mechanical strength and water vapor barrier properties. Fewer pores and uniform polymer phases reduce moisture pathways and enhance load-bearing capacity [55]. In this study, the semi-crystalline–amorphous structures formed under dry heat treatment align with the findings of Antony Samy et al. [56], who highlighted structural integrity as a key factor for film stability. Thus, SEM confirms that thermal modification promotes starch chain rearrangement, leading to stronger film structures.

### 3.11. Surface Morphology of the Optimized Film by Atomic Force Microscopy (AFM)

**Figure 10:**
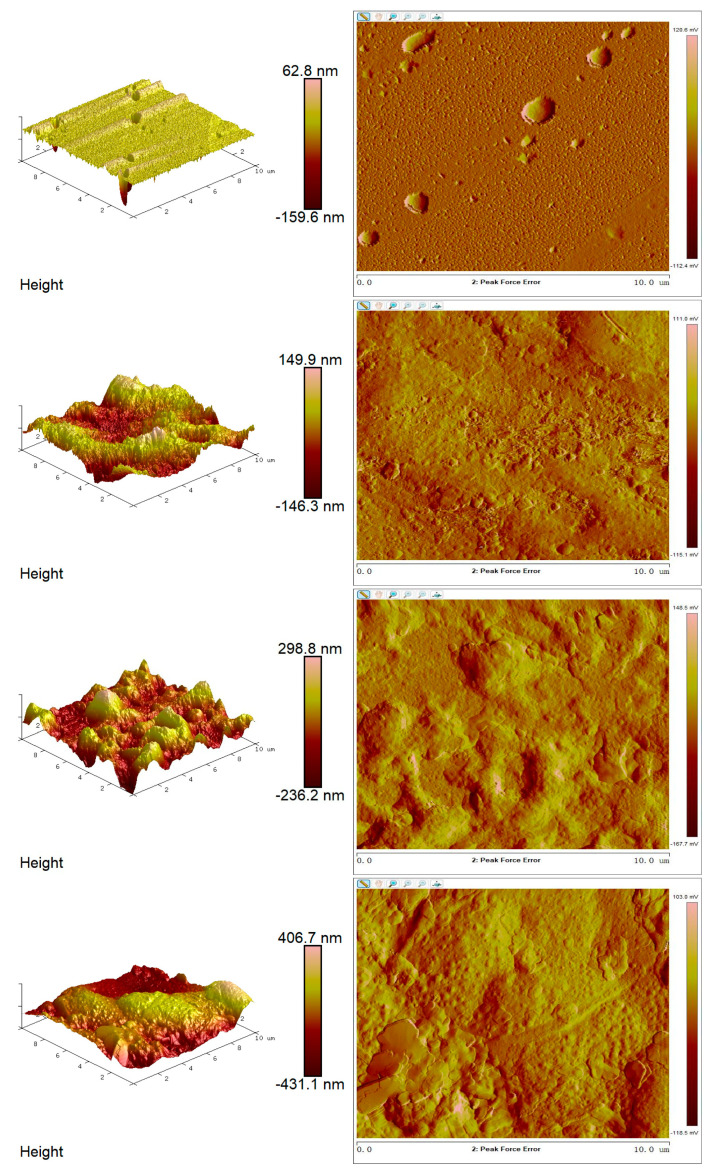
AFM surface topography of the optimized edible antimicrobial film, demonstrating uniform morphology and low surface roughness with height variations between 62.8 and 406.7 nm.

Atomic force microscopy (AFM) analysis provided detailed insights into the surface morphology of the optimized edible antimicrobial film. As shown in Figure 10, the film exhibited a continuous, homogeneous, and compact surface. The height variations ranged from 62.8 nm to 406.7 nm, indicating that the film surface exhibited low to moderate micro-roughness.

Importantly, the absence of large pores or cracks on the surface of the optimized samples suggests high structural uniformity and compatibility with standard film-forming processes. The relatively smooth surface topography may enhance the film’s barrier properties, including light transmittance, gas permeability, and water vapor resistance.

AFM analysis shows that surface roughness can influence film optical properties, contact angle, and gas barrier performance. Surface irregularities increase light scattering and may enhance moisture or oil permeability through capillary effects [57]. In this study, moderate roughness (62.8–406.7 nm) corresponded with a water contact angle of 84.6°, indicating a hydrophobic and structurally stable surface. Muscat et al. [58] also noted that the mixed phase of amylose and amylopectin affects film wettability. Thus, AFM confirms the tunable surface properties of films formed from thermally modified starch.

### 3.12. Optical Color Properties of the Optimized Film by Laser Analysis

Laser-based colorimetric analysis (Table 8) revealed that the optimized edible film samples exhibited high color uniformity and visual stability. As shown in Table 9, the L* values ranged from 89.45 to 90.82 across all samples, indicating high brightness and a light-toned appearance. The a* and b* coordinates remained near neutral, with a* values between 0.15 and 0.31 and b* values between −0.16 and 0.40, suggesting minimal chromatic deviation.

These results confirm that the film surface possesses homogeneous optical characteristics and is visually compatible for use in food packaging applications. The absence of significant fluctuations in color parameters implies that the film’s aesthetic quality can be consistently maintained during repeated manufacturing processes.

In summary, the laser-based color analysis confirmed the high aesthetic quality of the optimized film and its potential to positively influence consumer perception in food packaging applications.

### 3.13. Antioxidant and Antibacterial Activities of Film Extracts

Bioactivity tests of water extracts derived from the optimized edible antimicrobial film confirmed both antioxidant and antibacterial potential. As shown Table 10 by DPPH and ABTS radical scavenging assays, the film extracts demonstrated radical inhibition rates of 25.94 ± 1.12%, 24.53 ± 1.07%, and 23.74 ± 1.14% (DPPH), and 25.23 ± 1.27%, 23.04 ± 1.32%, and 25.03 ± 1.22% (ABTS) for samples 1, 2, and 3, respectively. These values indicate the presence of natural antioxidant compounds within the film matrix, likely associated with chitosan and possible Maillard reaction products formed during dry heat modification, which are known to donate protons to neutralize free radicals.

Antibacterial activity was assessed in liquid nutrient media by adding film extracts and measuring bacterial growth inhibition through OD_600_ absorbance. All three samples showed substantial inhibitory effects. Specifically, *Escherichia coli* inhibition was 47.16 ± 1.54%, 46.88 ± 1.41%, and 55.14 ± 1.78%, while *Staphylococcus aureus* inhibition was 44.71 ± 1.89%, 38.31 ± 2.03%, and 32.12 ± 2.17%, respectively. These results demonstrate that the optimized starch-chitosan films are effective against both Gram-negative and Gram-positive bacteria, supporting their potential use in active food packaging systems.

The antioxidant activity of the film may result from residual amino groups in chitosan and potential phenolic compounds in dry heat–modified starch [59]. Both DPPH and ABTS assays confirmed its free radical scavenging ability, supporting oxidative stability in food. The antimicrobial effect is attributed to chitosan’s cationic nature under mildly acidic conditions, which disrupts bacterial membranes [60]. Moreover, the chitosan concentration used aligns with the optimal antimicrobial range reported by Hosseinnejad et al. [61]. Thus, the film offers not only physical protection but also bioactive functionality, making it suitable for active food packaging.

## 4. Conclusions

This study demonstrated the feasibility of using dry heat–modified starches from Kazakhstan as functional biopolymers for the development of edible antimicrobial films. Through a systematic investigation of the physicochemical properties of various native and thermally modified starches, corn and cassava starches were identified as the most suitable base materials for film formation. The incorporation of chitosan and glycerol further enhanced the films’ mechanical performance, barrier properties, and bioactivity.

By applying response surface methodology, the formulation process was optimized, resulting in a biodegradable film with good transparency, flexibility, and structural integrity. Microscopic and thermal analyses confirmed the uniformity and stability of the film matrix. In addition to its favorable physical properties, the film exhibited antioxidant capacity and antibacterial activity against common foodborne pathogens, indicating its potential application in active food packaging systems.

While these findings are promising, further studies are warranted to assess the film’s performance under real storage conditions and its compatibility with diverse food matrices. Exploring additional natural additives and evaluating biodegradability in practical environments would help expand the film’s applicability.

Overall, this work not only expands the understanding of Kazakhstani starch modification but also offers a practical strategy for the design of sustainable, bioactive films that contribute to food quality preservation and environmental protection.

## Figures and Tables

**Table 1:** Raw materials and their production regions.

Raw Material	Variety	Production Region
Potato	Gala	East Kazakhstan
Wheat	Steklovidnaya-24	North Kazakhstan
Corn	Altyn Dan	Almaty Region
Pea	Zhambyl 8	Zhambyl Region
Rice	Marzhan	Kyzylorda Region
Cassava	Cassava 531	South Kazakhstan

**Table 2:** Changes in gelatinization transparency of different starch types during storage at 650 nm.

No	Starch Type	Initial Transparency	24 h	48 h	72 h
1	Wheat starch	0.96 ± 0.08 ^a^	0.58 ± 0.04 ^b^	0.29 ± 0.02 ^c^	0.36 ± 0.09 ^c^
2	Corn starch	0.76 ± 0.03 ^b^	0.63 ± 0.05 ^a^	0.26 ± 0.07 ^c^	0.65 ± 0.03 ^a^
3	Potato starch	0.44 ± 0.05 ^c^	0.15 ± 0.06 ^d^	0.85 ± 0.04 ^a^	0.49 ± 0.01 ^b^
4	Rice starch	0.24 ± 0.06 ^d^	0.32 ± 0.03 ^c^	0.28 ± 0.02 ^c^	0.27 ± 0.04 ^c^
5	Cassava starch	0.24 ± 0.01 ^d^	0.97 ± 0.08 ^a^	0.91 ± 0.07 ^a^	0.89 ± 0.05 ^a^
6	Pea starch	0.33 ± 0.05 ^cd^	0.29 ± 0.04 ^c^	0.26 ± 0.03 ^c^	0.23 ± 0.07 ^c^

Note: Values are presented as mean ± standard deviation (n = 3). Different superscript letters (a, b, c, d) within the same column indicate significant differences among starch types at the same storage time (*p* < 0.05), according to Duncan’s multiple range test.

**Table 3:** Supernatant separation (%) of different starch types during three freeze–thaw cycles.

No	Starch Type	1st Cycle (%)	2nd Cycle (%)	3rd Cycle (%)
1	Wheat starch	67.04 ± 2.16 ^a^	61.97 ± 4.14 ^a^	22.74 ± 1.68 ^c^
2	Corn starch	53.92 ± 3.45 ^b^	47.65 ± 2.88 ^b^	20.91 ± 1.26 ^c^
3	Potato starch	32.47 ± 2.73 ^c^	13.49 ± 2.82 ^d^	59.95 ± 3.43 ^a^
4	Rice starch	19.36 ± 2.17 ^d^	11.24 ± 3.37 ^d^	28.25 ± 4.86 ^bc^
5	Cassava starch	28.17 ± 2.31 ^c^	18.02 ± 1.94 ^cd^	35.47 ± 2.76 ^b^
6	Pea starch	45.92 ± 1.56 ^b^	52.48 ± 3.06 ^b^	37.63 ± 2.41 ^b^

Note: Values are expressed as mean ± standard deviation (n = 3) and are presented in percent (%). Different superscript letters within the same column indicate statistically significant differences among starch types for each freeze–thaw cycle (*p* < 0.05), based on Duncan’s multiple range test.

**Table 4:** In vitro digestibility parameters of native and thermally modified starches.

Starch Type	Rapidly Digestible Starch (RDS, %)	Slowly Digestible Starch (SDS, %)	Resistant Starch (RS, %)
Cassava	52.0 ± 1.5 ^a^	24.0 ± 1.2 ^c^	24.0 ± 1.4 ^c^
Cassava (modified)	40.0 ± 1.3 ^b^	28.0 ± 1.0 ^b^	32.0 ± 1.2 ^b^
Corn	48.0 ± 1.4 ^a^	26.0 ± 1.1 ^bc^	26.0 ± 1.3 ^c^
Corn (modified)	39.0 ± 1.2 ^b^	29.0 ± 1.0 ^b^	32.0 ± 1.1 ^b^
Potato	45.0 ± 1.6 ^ab^	32.0 ± 1.2 ^a^	23.0 ± 1.5 ^c^
Potato (modified)	36.0 ± 1.4 ^b^	33.0 ± 1.1 ^a^	31.0 ± 1.3 ^b^
Pea	42.0 ± 1.3 ^ab^	27.0 ± 1.0 ^bc^	31.0 ± 1.2 ^b^
Pea (modified)	33.0 ± 1.2 ^c^	33.0 ± 1.0 ^a^	34.0 ± 1.4 ^ab^
Rice	35.0 ± 1.5 ^c^	31.0 ± 1.1 ^ab^	34.0 ± 1.3 ^ab^
Rice (modified)	28.0 ± 1.3 ^c^	30.0 ± 1.0 ^ab^	42.0 ± 1.2 ^a^
Wheat	41.0 ± 1.4 ^ab^	28.0 ± 1.0 ^b^	31.0 ± 1.1 ^b^
Wheat (modified)	34.0 ± 1.2 ^b^	30.0 ± 1.0 ^ab^	36.0 ± 1.3 ^ab^

Note: Values are expressed as mean ± standard deviation (n = 3). Different superscript letters within the same column indicate statistically significant differences among starch types (*p* < 0.05), according to Duncan’s multiple range test.

**Table 5:** Factor levels used in the Box–Behnken design for the preparation of edible antimicrobial films.

Level	Starch Content A (%)	Gelatinization Time B (min)	Glycerol Content C (%)	Chitosan Content D (%)
−1	1.0	20	1.0	0.5
0	3.0	30	3.0	1.5
+1	5.0	40	5.0	2.5

**Table 6:** Box–Behnken experimental design matrix and measured responses for edible antimicrobial films.

Run Order	A (%)	B (min)	C (%)	D (%)	Std Order	Tensile Strength (MPa)	Elongation at Break (%)	WVP (g/m^2^/day)	Transparency (%)
1	0	0	1	−1	6	10.55	45.2	261.9	73.2
2	−1	0	0	−1	9	10.23	48.3	277.3	68.0
3	−1	−1	0	0	1	10.62	46.3	282.4	72.5
4	−1	1	0	0	3	11.06	46.9	281.2	73.9
5	0	0	−1	1	7	10.18	46.3	259.9	76.7
6	−1	0	0	1	11	10.18	51.2	266.3	71.7
7	1	0	0	−1	10	11.09	47.5	274.0	71.0
8	1	1	0	0	4	10.68	45.4	281.7	71.7
9	0	−1	0	−1	21	10.07	49.1	264.2	75.3
10	0	0	−1	−1	5	10.57	45.1	267.8	73.8
11	1	0	1	0	20	10.07	47.9	256.7	71.7
12	0	0	0	0	25	10.42	43.6	255.6	74.3
13	0	−1	0	1	23	9.34	44.8	279.8	73.2
14	0	0	0	0	26	9.44	47.9	286.3	75.4
15	1	0	0	1	12	10.02	49.0	269.1	71.2
16	0	0	0	0	27	9.79	47.8	282.0	72.2
17	0	−1	1	0	15	10.46	47.3	274.3	72.0
18	−1	0	1	0	19	9.85	46.9	262.3	69.3
19	1	−1	0	0	2	9.59	44.5	274.3	73.7
20	0	1	1	0	16	11.03	46.1	288.5	73.7
21	0	1	0	−1	22	10.19	46.6	269.6	73.0
22	1	0	−1	0	18	10.33	49.6	288.8	72.4
23	0	1	−1	0	14	9.59	48.2	238.6	69.5
24	0	0	1	1	8	10.03	44.0	279.9	71.9
25	−1	0	−1	0	17	10.03	48.1	271.0	72.1
26	0	1	0	1	24	10.36	46.7	266.4	71.0
27	0	−1	−1	0	13	10.22	45.4	264.9	70.2

Note: A: Starch concentration (%), B: Gelatinization time (min), C: Glycerol concentration (%), D: Chitosan concentration (%). WVP: Water Vapor Permeability (g/m^2^/day).

**Table 7:** Optimal combination of formulation parameters for edible antimicrobial film.

Factor	Coded Level	Actual Value (Interpolated)
Starch concentration (A)	+1.0	≈5.0%
Gelatinization time (B)	−0.1	≈28.2 min
Glycerol concentration (C)	−0.2	≈2.8%
Chitosan concentration (D)	−0.1	≈1.4%
Predicted Transparency (%)	–	~77.5%

**Table 8:** Physico-mechanical and structural properties of the optimized edible antimicrobial film.

Property	Average Value
Film thickness (mm)	0.0492 mm
Transparency (A_600_)	1.205
WVTR (g/m^2^·day)	270.34 g/m^2^·day
WVP (g/m·s·Pa)	6.652 × 10^−10^ g/m·s·Pa
Water contact angle (°)	84.6°
Tensile strength (MPa)	10.92 MPa
Elongation at break (%)	50.0%

**Table 9:** Colorimetric properties of optimized edible antimicrobial film samples determined by laser analysis.

Sample	Mode	Illuminant/Angle	REF_XYZ	L*	a*	b*
KZ-1	SCI	C/2°	0	88.18 ± 0.42 ^c^	0.31 ± 0.02 ^a^	0.34 ± 0.03 ^a^
KZ-2	SCI	C/2°	0	90.82 ± 0.35 ^a^	0.27 ± 0.01 ^b^	−0.16 ± 0.02 ^d^
KZ-3	SCI	C/2°	0	89.63 ± 0.38 ^b^	0.26 ± 0.01 ^b^	−0.06 ± 0.02 ^c^
KZ-4	SCI	C/2°	0	90.74 ± 0.36 ^a^	0.30 ± 0.01 ^a^	−0.12 ± 0.02 ^b^

Note: L* represents brightness (0 = black, 100 = white); a* represents green (−) to red (+); b* represents blue (−) to yellow (+); Values are expressed as mean ± standard deviation (n = 3). Different superscript letters within the same column indicate statistically significant differences among samples at *p* < 0.05, based on Duncan’s multiple range test.

**Table 10:** Antioxidant and antibacterial activities of water extracts from optimized edible antimicrobial films.

Sample	DPPH (%)	ABTS (%)	*E. coli* Growth Inhibition (%)	*S. aureus* Growth Inhibition (%)
1	25.94 ± 1.12 ^e^	25.23 ± 1.27 ^a^	47.16 ± 1.54 ^d^	44.71 ± 1.89 ^f^
2	24.53 ± 1.07 ^b^	23.04 ± 1.32 ^c^	46.88 ± 1.41 ^e^	38.31 ± 2.03 ^g^
3	23.74 ± 1.14 ^c^	25.03 ± 1.22 ^b^	55.14 ± 1.78 ^c^	32.12 ± 2.17 ^e^

Note: Values are expressed as mean ± standard deviation (n = 3). Different superscript letters within the same column indicate statistically significant differences among samples at *p* < 0.05, based on Duncan’s multiple range test.

## Data Availability

The original contributions presented in this study are included in the article. Further inquiries can be directed to the corresponding authors.

## Abbreviations

The following abbreviations are used in this manuscript:

RSM	Response Surface Methodology
BBD	Box—Behnken Design
WVP	Water Vapor Permeability
SEM	Scanning Electron Microscopy
AFM	Atomic Force Microscopy
DSC	Differential Scanning Calorimetry
TGA	Thermogravimetric Analysis
FTIR	Fourier Transform Infrared Spectroscopy
RVA	Rapid Visco Analyzer
DPPH	2,2-Diphenyl-1-picrylhyd razyl
UV	Ultraviolet
CFU	Colony-Forming Units
TS	Tensile Strength
EAB	Elongation at Break
ANOVA	Analysis of Variance
L*, a*, b*	CIE Color Space Coordinates
